# BRAF V600-Mutated Metastatic Melanoma and Targeted Therapy Resistance: An Update of the Current Knowledge

**DOI:** 10.3390/cancers15092607

**Published:** 2023-05-04

**Authors:** Laetitia Florent, Charles Saby, Florian Slimano, Hamid Morjani

**Affiliations:** 1 Université de Reims Champagne-Ardenne, UFR de Pharmacie, BioSpecT EA 7506, 51097 Reims, France; laetitia.florent.etu@gmail.com (L.F.); charles.saby@univ-reims.fr (C.S.); florian.slimano@univ-reims.fr (F.S.); 2 CHU Reims, Department of Pharmacy, 51097 Reims, France

**Keywords:** melanoma, mutated BRAF, targeted therapies, signal transduction, microenvironment, resistance

## Abstract

**Simple Summary:**

Metastatic melanoma is the most lethal skin cancer due to its high metastatic potential and resistance to treatment. The advent of targeted therapies has improved patient management. However, due to the emergence of resistance shortly after the start of treatment, the overall and progression-free survival remain limited. In this review, we aim to update recent published data about metastatic melanoma, its therapeutic management, and the associated resistance mechanisms involved in disease relapse. We describe recent data on cellular and microenvironment-induced resistance to targeted therapies. The discovery of targetable mutations and the understanding of the mechanisms involved in the development of metastatic melanoma has allowed for the improvement in patient treatments thanks to targeted therapies and immune checkpoint inhibitors. Further understanding of other non-elucidated mechanisms of resistance will contribute to improving melanoma patients’ management.

**Abstract:**

Melanoma is the most common cause of death in skin cancer due to its high metastatic potential. While targeted therapies have improved the care of patients with metastatic melanoma harboring the BRAFV600E mutation, these treatments are associated with a high frequency of resistance. Resistance factors are related to cellular adaptation as well as to changes in the tumor microenvironment. At the cellular level, resistance involves mutations, overexpression, activation, or inhibition of effectors involved in cell signaling pathways such as MAPK, PI3K/AKT, MITF, and epigenetic factors (miRNAs). In addition, several components of the melanoma microenvironment, such as soluble factors, collagen, and stromal cells also play a crucial role in this resistance. In fact, extracellular matrix remodeling impacts the physical and chemical properties with changes in the stiffness and acidity, respectively of the microenvironment. The cellular and immune components of the stroma are also affected, including immune cells and CAF. The aim of this manuscript is to review the mechanisms responsible for resistance to targeted therapies in BRAFV600E-mutated metastatic melanoma.

## 1. Melanoma Development

Skin cancers include non-melanoma skin cancers (basal cell skin carcinomas and squamous cell skin carcinomas) and melanoma cancers. Non-melanoma skin cancers have a good prognosis and are treated via surgical excision. Despite the low prevalence of melanoma among skin cancers, it remains the main cause of death from skin cancer. According to the WHO, in 2020, non-melanoma cancers worldwide had an incidence of 1,198,073, with a mortality of 63,731, whereas the incidence of melanoma was lower, at 324,635, with a mortality almost equivalent to non-melanoma cancer, at 57,043.

The origin of melanoma comes from the malignant transformation of melanocytes (Table 1), a low-dividing cell population localized in the basal layer of the epidermis and in hair follicles whose main function is to produce melanin to the surrounding keratinocytes. Melanoma development could be governed by driver mutations such as BRAF, H/N/K-RAS, and C-KIT and can be associated with secondary genetic or epigenetic alterations that may affect tumor suppression pathways. The BRAFV600E mutation was first described in 2002 when a study from Davies et al. revealed the existence of BRAF-mutated proteins in about 66% of malignant melanomas and at a lower frequency in other types of human cancers. This mutation results in a much higher kinase activity than the wild-type protein and confers to the cells an independence from the RAS protein function, as well as a transforming activity and a tenfold increase in cell growth. At that time, their conclusion proposed this mutated protein as a potential therapeutic target, and this allowed for the modern method of targeting mutations in melanoma [1]. In 2015, researchers from the Cancer Genome Atlas Network (TCGA) classified melanoma into four major molecular subtypes: BRAF, RAS (N/H/K), NF1, and Triple-WT, with the first three subtypes resulting in a permanent activation of the MAPK/ERK [2]. This results in the activation of key oncogenic pathways leading to an increase in melanoma cell proliferation, invasion, and metastatic abilities. Approximately 50% of melanoma exhibited BRAFV600 mutations. Up to 90% of BRAFV600 mutations correspond to a substitution of valine by glutamic acid at codon 600 (BRAFV600E) [3]. The second most common mutation is the V600K mutation (about 5–6%), which is thought to be more prevalent in certain populations, such as the Australian population [4]. The other mutations (including BRAFV600R and BRAFV600D) are very rare [5]. BRAF is a serine/threonine protein kinase which is encoded by the gene BRAF located on chromosome 7 that activates the MAP kinase/ERK signaling pathway. The mutation BRAFV600E causes a constitutive activation of the kinase combined with a suppression of the negative feedback, leading to the permanent activation of the downstream MEK/ERK pathway. This process increases cell proliferation, promotes tumor cell invasion, and facilitates metastasis [5].

The main risk factors are a personal or family history of melanoma and high exposure to ultraviolet (UV) radiation, either from sunlight or from the use of tanning booths. The risk is also increased for people who sunburn easily or who have a history of excessive sun exposure [6]. That is why melanoma tumors are most often located on sun-exposed skin [7]. Melanoma tumors present a large inter-individual variability that is related to the anatomical site of the primary tumor, the degree of exposure to ultraviolet radiation, the age of the patient, and the charge and type of the oncogenic mutations [7]. Most melanomas develop in the skin and can be classified into four categories: extensive superficial cutaneous melanomas (the most common), nodular melanomas, lentigo maligna, and acral lentiginous melanomas. In addition, some melanomas develop in the mucous areas (oral, ano-genital, ocular, visceral); these forms have a poor prognosis because they are often diagnosed late. In 2018, the World Health Organization (WHO) identified nine pathways leading to melanoma, with each having specific genetic signatures. Melanocytic lesions are dissociated into three broad categories based on cumulative solar damage (CSD) [8]. TERT and CDKN2A, PTEN, KIT, and TP53 mutations are frequently observed in melanoma tumors; moreover, the number of possible mutation types increases with CSD status, thus High-CSD melanomas have the highest tumor mutation burden. The category of melanomas with “little or no UV/CSD exposure” rarely harbor BRAF, NRAS, or NF1 mutations [9].

**Table 1:** Common melanoma progression classification. Data from Shain and Bastian, 2016 [7].

Naevus	Intermediate Neoplasm	Melanoma In Situ	Invasive Melanoma (Metastatic or Unresectable)
Result of benign proliferation of melanocytes; very low probability of developing into melanoma. The number and size are influenced by the frequency of initiating mutations, such as the BRAFV600E mutation which is frequent in nevi.	Melanocytic neoplasms containing lesions with overlapping benign and malignant histopathological features.	Large-nucleated melanocytes that grow irregularly entirely within the epidermis. Survival rate = nearly 100% for completely resected melanoma in situ.	Cancer cells have left the epithelium of the epidermis to move into the underlying mesenchymal tissue, (dermis /submucosa). The risk of death correlates with the extent of invasion.

The staging system for cutaneous melanoma is based on the evaluation of the parameters T (thickness of the primary tumor, presence, or absence of ulceration), N (number of lymph nodes involved and presence or absence of metastases), and M (anatomical site of distant metastases and LDH levels). Thus, the patient’s stage of progression is defined as 0; I (IA and IB); II (IIA, IIB and IIC); III (IIIA, IIIB, IIIC, and IIID); or IV. In stages 0, I, and II, the tumor is localized, while in stages III and IV there is involvement of lymphatic tissue and dissemination to one or more vital organs [10].

## 2. Melanoma Treatment

Melanoma treatment is based on the combination of the four major approaches: surgery, radiotherapy, anticancer treatment, and best supportive care. At an early stage, melanoma can be successfully treated via surgical removal. However, when tumor cells invade the dermis, this type of treatment is no longer effective. Until the 2010s, metastatic melanoma had very poor therapeutic options. The previous treatments were based on the use of alkylating agents that affect cell division by inducing DNA damage in a non-selective way (dacarbazine and fotemustine) with poor survival outcome. Dacarbazine allowed for the obtaining of a very low overall survival, of 5.6 to 7.8 months, after the beginning of the treatment [11]. Fotemustine was used preferentially when central nervous system metastasis was detected at diagnosis. In 2011, two major clinical trials opened the new era of melanoma treatment with significant improvement in overall survival.

- The BRIM3 clinical trial shows the superiority of vemurafenib (a kinase Braf protein inhibitor) over dacarbazine in the treatment of BRAFV600E-mutated melanoma, with a 3.6 month gain in overall survival [12].
- The NCT00324155 clinical trial shows that ipilimumab (a monoclonal antibody blocking cytotoxic T-lymphocyte-associated antigen 4, CTLA4) in combination with dacarbazine improves overall survival compared to dacarbazine alone, with a 2.1 month gain in overall survival [13].

**Figure 1:**
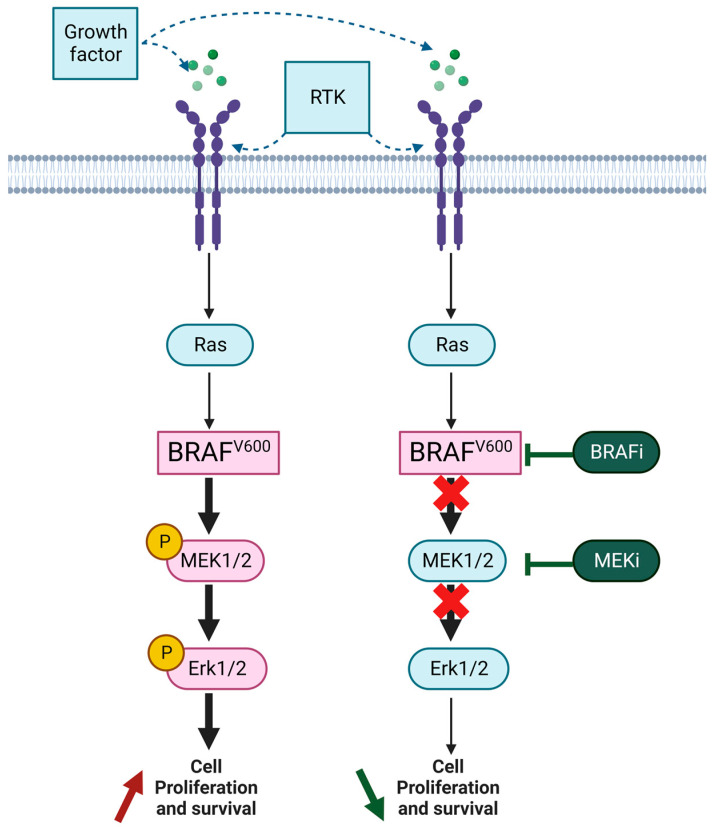
Schematic representation of alterations in the RAS-BRAF-MEK-ERK MAP kinase signaling pathway with V600-mutated BRAF and sites of therapeutic kinase inhibition. BRAFi: BRAF inhibitor; MEKi: MEK inhibitor.

These clinical trials have opened up therapeutic opportunities for two major classes of therapies: targeted therapies and immune checkpoint inhibitors (ICIs). In melanoma, the BRAF mutation leads to a constitutive activation of the BRAF-MEK-ERK pathway, leading to increased proliferation and cell growth. The aim of targeted therapies is to inhibit the signaling cascade of the protein kinase activated by mitogenic agents (MAPKs) (Figure 1). To this end, inhibitors of this mutated kinase have been developed and then associated with MEK inhibitor downstream of the BRAF protein, giving the combinations: vemurafenib/ cobimetinib, dabrafenib/ trametinib or encorafenib/ binimetinib [14]. ICIs promote immune antitumor activity. Ligands CTLA4 (cytotoxic T-lymphocyte-associated protein 4) and PD-1 (programmed cell death 1) located on T lymphocytes are negative regulators of immune anti-tumor activity when bound to their receptors B7 on dendritic cells (for CTLA4) and PD-L1 (PD-Ligand 1) on tumor cells (for PD-1). The identification of this mechanism led to the development of monoclonal antibodies that bind PD-1 (Nivolumab and Pembrolizumab), PD-L1 (atezolizumab), and CTLA4 (ipilimumab). First alone and then in combination, anti-PD1 and anti-CTLA4 are associated with a dramatical increase in progression-free and overall survival [10,15].

The resection of melanoma in its early stages is in most cases sufficient to be curative. However, for more advanced stages, the resected melanoma presents a high risk of relapse. To avoid this phenomenon, adjuvant therapies have been established [16]. Thus, immunotherapies such as pembrolizumab and nivolumab have been approved for the treatment of stage IIB, IIC, or III melanoma after complete resection. In addition, the BRAF and MEK inhibitor combination dabrafenib/trametinib has received approval for the treatment of stage III melanoma after complete resection when it presents a BRAF mutation [17]. Clinical trials are ongoing for the use of these therapies in a neoadjuvant indication, with positive preliminary results [18].

**Figure 2:**
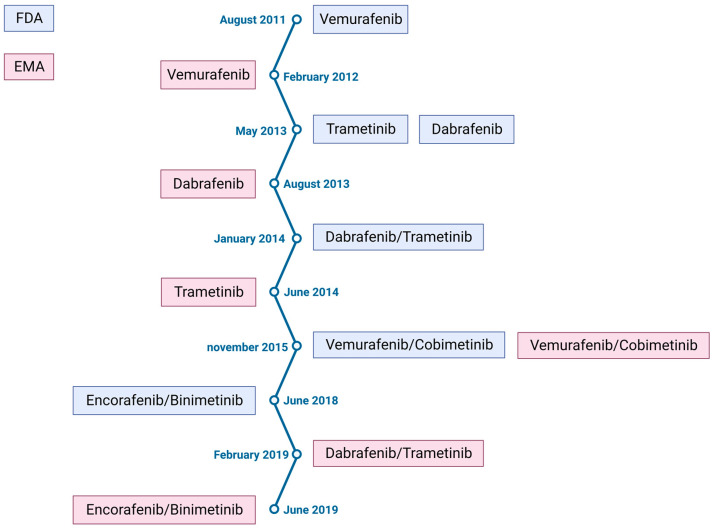
Chronology of the approval of targeted therapies by the Food and Drug Administration (FDA) and by the European Medicines Agency (EMA).

For metastatic melanoma with a BRAF mutation, the advent of targeted bi-therapies developed as a result of these early clinical trials has improved progression-free survival and overall survival for patients [19] (Table 2 and Figure 2). Melanomas harboring other mutations, such as RAS mutation or non-V600 BRAF mutations, do not respond to BRAF inhibitors. This is mostly the case for mucosal melanoma, as the BRAF mutation is less frequent in this type of melanoma [20]. Nowadays, the first-line treatment for advanced melanoma is mainly based on immune checkpoint inhibitors alone (anti-CTLA4 or anti-PD1) or in combination (anti-CTLA4 and anti-PD1) [21]. However, with consideration for the BRAF mutation status, especially in the case of rapid disease progression, melanoma will be treated in first line with the combination of a BRAF inhibitor and a MEK inhibitor. These inhibitors may also be used in second line if not previously used. [22]. In addition, studies are underway to combine ICIs and targeted therapies, either concomitantly [23,24] or sequentially [25]; the results are mainly positive, but no approval has yet been obtained.

Targeted therapies and immune checkpoint inhibitors are now part of the therapeutic strategy depending on the cancer stage and BRAF mutational status. Targeted therapies are particularly effective in aggressive melanoma with great clinical response. Even with high response rates (approximately 70%), some patients remain completely unresponsive to such therapy. Among patients with good response to anti-angiogenic treatments, many of them are refractory to these therapies due to the emergence of resistance [26,27]. Moreover, this resistance may occur several months after the beginning of treatment and is characterized by a disease relapse after partial or total remission [14]. These observations lead us to address the following question: What are the mechanisms involved in the resistance of BRAFV600E-mutated melanoma to targeted therapies? In this review we will focus on the resistance due to alteration of signaling actors involved in the BRAF pathway, before discussing the impact of the microenvironment on the response to these therapies.

**Table 2:** Impact of targeted therapies on metastatic melanoma’s progression-free and overall survival.

Anti-BRAF	Vemurafenib	Dabrafenib	Encorafenib
Anti-MEK	Cobimetinib	Trametinib	Binimetinib
Efficacity	2011: trial coBRIM NCT03224208 [28] Median PFS: 12.6 months PFS rate: 14% at 5 years Median OS: 22.5 months OS rate: 31% at 5 years	2012: trial COMBI-d NCT01584648 [29] Median PFS: 11 months PFS rate: 19% at 5 years Median OS: 25.9 months OS rate:34% at 5 years	2018: trial COLUMBUS NCT01909453 [30] Median PFS: 14.9 months PFS rate: 23% at 5 years Median OS: 33.6 months OS rate: 35% at 5 years

## 3. Cellular Resistance

**Figure 3:**
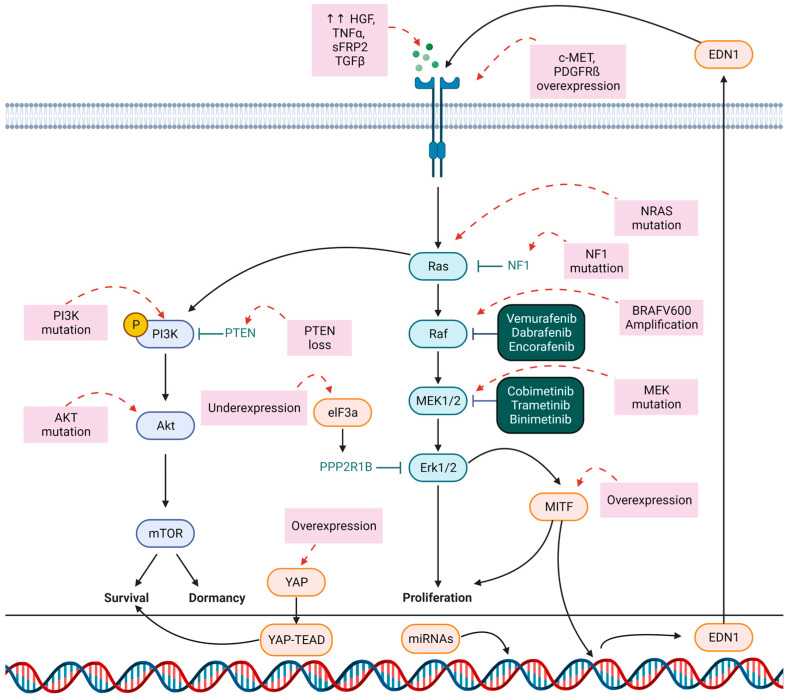
Schematic representation of the main pathways involved in melanoma cell resistance to targeted therapies.

There are commonly two mechanisms of resistance to BRAF/MEK inhibitors: primary (intrinsic) and secondary (acquired) resistance. Primary resistance occurs when patients do not respond to the initial therapy according to the Response Evaluation Criteria in Solid Tumors (RECIST) criteria [31]. It affects approximately 15% of patients with mutated BRAF and can be explained by the activation of substitutive pathways: for example, a bypass pathway such as the upregulation of the PI3K/AKT/mTOR pathway. Increased PI3K activity can be caused by the loss of tumor suppressors such as PTEN phosphatase or neurofibromin 1 (NF1) [14]. Secondary resistance occurs several weeks/months after the start of BRAF/MEK inhibitors, after an initial response to target therapies (complete or partial remission). One year before the approval of vemurafenib in the United States and Europe, studies have already highlighted resistance to targeted therapies. In fact, the rapid and significant efficacy of BRAF inhibitors is temporary. Resistance emerges in most cases and occurs through various cellular mechanisms, including the reactivation of MAPKs (for example with overexpression of N-RAS) or activation of alternative receptor tyrosine kinase (RTK)-mediated survival pathways (overexpression of PDGFRβ for example) (Figure 3) [32].

A 2015 study realized on 132 samples obtained from tissues of patients in the beginning stages of progression treated with BRAF inhibitors identifies mechanisms potentially involved in resistance in 58% of cases. They include NRAS/KRAS mutations in 20% of cases, with BRAF variants or BRAF amplifications in 30% of cases, and a MEK1/2 mutation in 7% of cases. In addition, alterations in other non-MAPK pathways have been reported in 11% of cases (PI3K/AKT, increased MITF, increased PDGFR/IGF1R) [33]. The PI3K signaling pathway is associated with resistance to BRAF and MEK inhibitors, but the mechanisms of activation for this pathway appear to follow very diverse patterns. For example, one study shows that PI3K/AKT activation does not preclude response to BRAF and MEK inhibition but promotes survival. This mechanism promotes resistance in surviving tumor cells and is associated with reactivation of MAPK but is no longer dependent on the initial oncogene activating PI3K/AKT. This form of resistance is dynamic and upgradeable, allowing the selection of highly resistant tumor clones, kept in dormancy by the activation of PI3K/AKT [34]. Another mechanism is related to the regulation of eIF-4F mRNA expression. It is composed of the binding protein eIF4E, the scaffolding protein eIF4G, and the RNA helicase eIF4A, and has been associated with resistance to BRAF pathway inhibitors. The eIF4F complex expression is increased in resistant metastases when compared to primitive tumors. Inhibition of this complex acts synergistically with BRAF inhibition [35]. In addition, a recent study has demonstrated that eIF3A, the largest subunit of the eIF3 complex, was a key factor behind resistance to BRAF inhibitors. It plays an important role in the interaction and recruitment of mRNA on the ribosome through the interaction between eIF3 and the eIF4G factor attached to the cap at the 5′ end of the mRNA. At the cellular level, it regulates cell cycle, apoptosis, differentiation, and fibrosis. The eIF3 complex has been reported to be expressed at lower levels in melanoma cells which are resistant to vemurafenib. Such a mechanism of resistance involves the control of ERK activity via a regulation of the PPP2R1B phosphatase expression by eIF3A at the translation level [36]. Interestingly, another mechanism would involve the Microphtalmia-associated transcription factor MITF. One of the processes of resistance to BRAF inhibitors is through an epithelial–mesenchymal transition (EMT) mechanism. This process is characterized by a MITF-low/AXL-high cell phenotype [37]. During relapse, there is in most cases a general tendency towards an AXL-directed transcriptional pathway, which theoretically leads to reduced MITF expression in these resistant tumors. In contradiction with this reasoning, 23% of relapsed melanomas show increased MITF expression, with two possible explanations: transcriptional plasticity, and MITF gene amplification correlated with disease progression. Eventually there would be intercellular communication thanks to the EDN1 factor secreted by MIFT-high cells in response to BRAFi, leading to the stimulation of the proliferation of AXL-high cells [38]. In most cases, BRAFi and MEKi resistance starts with changes in cell phenotypes, leading to therapeutic tolerance. Drug tolerance is defined as a progressive decrease in sensitivity to the effects of a drug resulting from its continuous administration [39]. Exposure to anticancer drugs induces a stress allowing the selection of subpopulations of cells that are proliferatively low and resistant to treatment. These so-called dormant cells may have their phenotype altered later and acquire a high proliferation rate and resistance phenotype [34]. Using a proteomic approach, it has been shown that even short-term exposure to BRAF inhibitors leads to an early state of cellular adaptation and differentiation; inhibition of the MAPK pathway results in the “proliferation” of melanoma cells, harboring at least partially the phenotypic characteristics of the “invasive” state. On the other hand, the treatment would induce a population of slow-cycling persistent cells that would later be a reservoir for the emergence of fully resistant proliferative cells [40]. Exposure to MAPKi can lead to chromatin changes and the upregulation of histone demethylases, which will select dedifferentiated, slow-cycling, drug-tolerant melanoma cells [38]. A study with a transcriptomic analysis showed that c-Met expression was increased to a supra physiological level in cohorts of patients with acquired resistance. Using Formalin-Fixed Paraffin-Embedded (FFPE) samples they detected the relative overexpression of c-MET protein in tissue sections in disease progression and found that c-MET overexpression was specific to MAPKi-resistant tumor cells. Assays on cell lines were performed and confirmed that the lines showing an overexpression of C-MET were resistant to MAPKi, in part related to an overactivation of AKT [41]. The same team performed a genetic signature analysis, and they observed that MAPKi-resistant cell lines had higher levels of the Yes-associated protein (YAP). This observation was confirmed via an analysis of progressive melanoma tissue compared to the reference tissue; an overexpression of YAP and pYAP was definitely present. In addition, knockdown of YAP in the resistant lines restored sensitivity to MAPKi. In summary, this comparative transcriptome analysis of matched melanoma tumor biopsies before and during progression highlights increased expression of c-MET and YAP as drivers of acquired resistance [41].

The impact of epigenetic factors such as microRNAs (miRNAs) needs to be taken into account because of their emerging roles in melanoma characteristics, including proliferation, migration, apoptosis, immune responses, and therapeutic resistance. Indeed, cancer-associated fibroblasts (CAFs), normal fibroblasts, and cancer cells establish an inter-cellular communication network in the tumor microenvironment through the secretion of miRNAs. Overexpression of miR-211 in melanoma cells induces their resistance to BRAF and MEK inhibitors, which is associated with an increase in the phosphorylated fraction of ERK5 effectors of a MAPK pathway other than BRAF. This effect is mediated through inhibition of the expression of DUSP6, a phosphatase of ERK5 [42]. In addition, a study on A375 melanoma cells resistant to vemurafenib reported overexpression of miR-204-5p, miR-211-5p in response to BRAFi treatment. The same effect was observed with MEKi treatment and the combination of BRAFi and MEKi. Pre-existing overexpression of these miRNAs results in primary resistance. Overexpression of these miRNAs appears to be mediated in part by activation of the transcription factors STAT and MITF. These miRNAs reactivate the MAPK pathways [43]. Conversely, the overexpression of miRNA 181a/b correlates with a better response to targeted therapies and the better survival of patients. Indeed, at the cellular level, transfection of MiR-181a and -181b mimics in A375 and M14-BIR cells, which are resistant to dabrafenib, and restored their sensitivity to the drug. The privileged target of MiR-181a and -181b seems to be the mitochondrial transcription factor A (TFAM), known to be an oncogenic regulator of mitochondrial gene expression and metabolism in melanoma. Their overexpression leads to TFAM silencing [44]. In the same way, the expression of miR-146a-5p is correlated with the sensitivity of melanoma to BRAF inhibitors. A decrease in the expression of this miRNA leads to an increase in Cox2 activity and NFkB signaling and resistance to BRAF inhibitors [45].

## 4. Microenvironment Mediated Drug Resistance

Acquired resistance to MAPK inhibitors in melanoma cannot be fully explained only by cellular mechanisms such as target mutation without taking into account the tumor microenvironment. In the microenvironment, cancer cells are confronted with different components, including extracellular matrix (ECM) adhesion proteins such as collagen, soluble factors, and stromal cells. The remodeling of the ECM components can impact tumor phenotype, invasion, and metastasis [46,47], but also the response to therapies [48,49]. In the case of melanoma, several related remodeling factors, such as ultraviolet radiation-related damages [50], collagen synthesis via fibroblasts, and aging have been reported to affect tumorigenesis and progression. Recent studies have highlighted that the ECM produced by resistant melanoma cells has topological and biochemical properties which are similar to those of ECM produced by tumor-associated fibroblasts. Such ECM is able on the one hand to protect melanoma cells against the anti-proliferative effects of BRAFV600E-targeted therapies [51]. On the other hand, melanoma cells harboring a mutational specific resistance phenotype to targeted therapies have been reported to induce ECM remodeling by upregulating the metalloproteinase MT1-MMP expression [52]. Indeed, analysis of RNA sequencing (RNAseq) data revealed an upregulation of MT1-MMP in relapsed melanoma after treatment. Inhibition of MT1-MMP by shRNA in resistant melanoma cells was able to restore their sensitivity to BRAF-targeted therapies. Furthermore, the increase in MT1-MMP expression in resistant melanoma cells was TGFβ-dependent. In fact, TGFβ receptor inhibition reduced MT1-MMP expression and restored sensitivity to BRAF-targeted therapies [52]. Interestingly, aging has been reported to impact melanoma metastasis and sensitivity to BRAF-targeted therapies. Indeed, Kaur et al. have shown that sFRP2 overexpression in aged dermal fibroblasts was able to increase the metastatic behavior and to decrease vemurafenib sensitivity in melanoma cells [53]. Furthermore, the subgroup analysis of the BRIM3 study that led to the marketing of vemurafenib showed a benefit in progression-free survival when compared to the alkylating agent dacarbazine in all age groups except for patients over 75 years [12].

These data support the hypothesis suggesting age as a factor of resistance to BRAF-targeted therapies. The extracellular microenvironment of solid tumors has been described to be acidic, and the low extracellular pH has been reported to promote resistance to vemurafenib in BRAFV600E melanoma cells [54]. More precisely, the acidic environment allows for the reactivation of the BRAF downstream pathway by increasing the MEK phosphorylation level. In addition, cells exposed to a low pH show an increase in the expression of the activated form of the ribosomal protein S6 kinase beta-1 (p70S6K), resulting in an increase in mTOR signaling. Accordingly, the mTOR inhibitor everolimus is able to overcome the resistance of BRAFV600E melanoma cells to BRAF and MEK inhibition [54]. Vascular remodeling is well known to contribute to the growth and resistance of melanoma.

A previous study has demonstrated that PI3K/AKT signaling can contribute to BRAF inhibitor resistance in melanoma [55]. Additionally, the insulin-like growth factor-1 (IGF-1R) can contribute to BRAF resistance to targeted therapies in melanoma. Such contribution has been reported to be due to the activation of AKT and ERK pathways, and subsequently the upregulation of E2F transcription factor 1 (E2F1) [56]. Interestingly, a study using a xenografted mice melanoma model has reported a vascular remodeling process which is mediated by the transduction signaling of the IGF1/IGF1R axis, contributing to the development of the melanoma cells which have acquired resistance to BRAF inhibition. These data provide a potential therapeutic strategy for the prevention of tumor relapse in clinic settings by combining a BRAF inhibitor and inhibition of IGF1/IGF1R axis [57].

Several factors produced by melanoma cells have been reported to promote the inflammation process [58,59], and inflammatory niches have been reported to adapt to and confer drug tolerance to BRAF and MEK inhibitors early during treatment. One of the proposed mechanisms in such a process is the amplification of inflammatory signaling in the presence of MAPK inhibitors. Indeed, melanoma cells can induce a pro-inflammatory phenotype in macrophages, resulting in an increase in IL-1β level. The recruitment of fibroblasts in such a process leads to a more complex crosstalk, which leads to the activation of IL-1 and subsequently the CXCR2 signaling pathways, leading to the secretion of several factors. Finally, these factors will increase melanoma cell survival by upregulating Bcl-2 expression. The inhibition of IL-1 receptor or CXCR2 signaling steps in association with the MAPK signaling pathway inhibition allows melanoma cells to overcome the resistance to BRAF inhibitors in vivo. Thus, a strategy aiming to interfere with both networks could lead to downregulation of the tumor growth and to delayed relapse in melanoma patients [60].

It is important to note that an initial treatment may itself have an impact on the sensitivity of the cells to future treatment. For example, synergy has been shown in the case of therapy combination involving targeted therapies and ICI. The combination of atezolizumab and BRAFi/MEKi-targeted therapies (vemurafenib/cobimetinib) results in an improved progression-free survival for patients compared with the treatment with only the double kinase-targeted therapy. This effect appears to be related to an increase in CD8+ cytotoxic T-cell proliferation [61]. However, a recent study highlighted a deleterious impact of targeted therapy in the case of relapse treatment with ICI. These observations are related to the modification of the immune environment by the inhibitors of BRAF kinase. Data showed particularly that acquired resistance to anti-MAPK-targeted therapy contributes to an immune-evasive tumor microenvironment and resistance to immunotherapy. Such resistance was related to a decrease in CD3+ and CD8+ T cell infiltration and CD103+ dendritic cell depletion in targeted therapy resistant tumors when compared to targeted therapy naive melanoma patients [62].

Furthermore, it has been shown that targeted therapies induce an increase in ECM rigidity in a YAP-dependent way. In response to targeted therapies, tumor cells acquire a CAF-like phenotype and consequently the ability to produce type I collagen, leading to an increase in stroma density and rigidity. Thus, this rigid collagen-rich matrix will confer resistance of melanoma cells to BRAF inhibitors [51]. The same group also showed that discoidin domain receptors DDR1 and DDR2, which are type I collagen receptors, are able to impact the melanoma cell phenotype. In fact, these receptors are able to regulate the cell proliferation, migration, invasion, and survival of several tumors [63,64,65]. Analysis of TCGA databases showed that a significant fraction of melanoma cells strongly expressed DDR1 and DDR2. This group has reported that targeting DDR1 and DDR2 was able to overcome collagen matrix-mediated tumor cell resistance to BRAF-targeted therapy in melanoma. [66]. A related recent work has shown that DDR2 receptor is overexpressed in the tumors of patients with secondary resistance to BRAF and MEK inhibitors. The authors highlighted two signaling pathways involved in the resistance to BRAF inhibitors. The first one involves RhoA activation, whereas the second one involves activation of the RAS/RAF/MEK/Erk pathway [37]. It has been shown that treatment with vemurafenib induces a “paradoxical” activation of melanoma-associated fibroblasts. Indeed, in response to the treatment, the activation of these fibroblasts is able to induce matrix remodeling by increasing collagen synthesis and modifying its topological organization. Such alterations induce in melanoma cells an activation of the integrin β1/FAK/Src pathway, which in turn activates the ERK pathway signaling and consequently leads to a resistance to BRAF inhibition. In this case, inhibition of Src or FAK overcomes the resistance phenotype [67].

## 5. Conclusions

Nowadays, the key to managing melanoma as a public health problem lies in raising awareness on the impact of UV radiation as the main cause of melanoma, as well as in early diagnosis. While targeted therapies have dramatically improved outcomes in metastatic melanoma management, it is important to note that they also encountered several limitations. The current challenge is to find combinations between recent treatments and new therapeutics while taking into account patient-related characteristics, such as the microenvironment remodeling occurring during aging. Many clinical trials are underway testing multiple possibilities, from sequential treatments (targeted therapies and immune checkpoint inhibitors) to the development of new nanotechnologies to propose more efficient drug nanocarriers. These nanotechnologies would allow for us to improve the pharmacokinetics of the drugs by localizing the distribution at the tumor area, thus making the treatment more effective with less side effects [10]. Finally, there is a new hope in the form of vaccines alone or in combination with immune checkpoint inhibitors in terms of boosting the immune system against the tumor [68].
